# Reverse Micelles Extraction of Prolamin from Baijiu Jiuzao: Impact of Isolation Process on Protein Structure and Morphology

**DOI:** 10.3390/ma17122901

**Published:** 2024-06-13

**Authors:** Ting-Ting Yu, Fu-Rong Yang, Yao Su, Yi-Heng Qi, Yi Liu, Nan Hu

**Affiliations:** College of Chemical Engineering, Sichuan University of Science and Engineering, 180 Xueyuan Road, Zigong 643000, China

**Keywords:** prolamins, Baijiu Jiuzao, reverse micelles extraction, secondary structure, protein structure

## Abstract

Prolamins, proteins derived from plants, have extensive applications in pharmaceutics and food science. Jiuzao is a byproduct of the Baijiu brewing industry, and is a great source of prolamin. Despite its importance, knowledge regarding the extraction techniques and the properties of prolamin derived from Baijiu Jiuzao (PBJ) remains limited. Reverse micelles (RMs) extraction offers an efficient and cost-effective method for purifying proteins. In the present study, prolamin was extracted from Baijiu Jiuzao using RMs extraction and subsequently characterized in terms of its secondary structure, morphology, and particle size distribution. Our findings indicate that the purified prolamin extracted using further RMs extraction possessed higher α-helix content (+13.25%), forming a large-scale protein network, and narrower particle size distributions compared to the crude prolamin obtained by NaOH-ethanol method. This research suggests that RMs extraction has potential applications in extracting prolamin from brewing industry byproducts, offering an environmentally friendly approach to Baijiu Jiuzao recycling.

## 1. Introduction

Prolamins are cereal proteins, which are parts of the storage protein found in plant seeds. The prolamin family includes zein (corn), kafirin (sorghum), gliadin (wheat), avenin (oat), secalin (rye), and hordein (barley) [1]. Prolamins, containing a high proportion of hydrophobic amino acids (proline, leucine, alanine) and some polar amino acids (glutamine), would be dispersed in alcoholic solutions [2,3]. Due to the low solubility in water, prolamins can be easily transformed into spherical colloidal nanoparticles by anti-solvent precipitation techniques, which makes it an ideal material to encapsulate active compounds [4,5]. Thus, prolamins could be applied to extending the shelf life of perishable food, stabilizing pickering emulsions, controlling release fertilizer and drug delivery systems [3,5]. As prolamins have shown outstanding hydrophobicity and film-forming ability, these proteins have attracted growing attention in the field of biodegradable polymeric nanoparticles.

Jiuzao is the by-product of baijiu production, which is the fermented residue of grains and rice husk. As one of the six most famous worldwide distilled beverages, Baijiu could be annually generated as high as ~3 × 10^11^ kg [6]. Besides the remarkably high production volumes of Baijiu, there is approximately 40 million tons of Jiuzao produced every year [7]. Because of the limitation and low efficiency of traditional distillation technology and solid-state fermentation, there are abundant proteins and fibers remaining in Jiuzao [7]. Jiuzao exhibits an acidic environment due to the organic acids produced by microorganisms such as yeast, acetic acid bacteria, molds, and bacteria [8]. If not properly managed, Jiuzao can pose environmental challenges, including air quality issues and soil contamination, with large-scale accumulations leading to foul odors and fly breeding [9]. At present, approaches to Jiuzao utilization can be majorly divided into four categories: (i) feeding, (ii) high-value component extraction, (iii) biomass energy production, and (iv) agriculture application (composting and soil conditioner) [7]. Nowadays, the majority of Jiuzao is merely used as a constituent of fodder for livestock [10]. The abundance of rice husks in Jiuzao results in poor feeding quality, ultimately contributing to a low price and small-scale consumption, thus leading to difficulties in making use of the large quantity of Jiuzao produced every year.

Despite rice husk, Jiuzao is the fermented residue of high-quality grains, such as sorghum, wheat, rice, corn, etc. These grains contain large amounts of prolamins. For example, around 70–90% of the total protein in sorghum [11] and approximately 80–85% of the total protein in wheat [12] is prolamins. During the fermentation process, large amounts of starch and saccharides are consumed while most prolamins are left over. Studies show that prolamins account for approximately 45% of the total protein of Jiuzao [13]. Studies show from 2014 and up to 2019, around 247 investigations have been published dealing with the obtention of nanoparticles and nanofibers using prolamins, of which only 2.0% correspond to materials obtained from cereals by-products [5]; even fewer reports are about prolamins obtained from Jiuzao. Recycling the prolamins in Jiuzao (PBJ) might be an efficient and environmentally friendly alternative strategy with economic value.

Studies show that prolamin molecules like kafirins from sorghum are polymeric and monomeric, easily forming a cross-linked aggregation. Alao prolamins tend to form more highly extended, strong web-like microstructures during the brewing processing [14]. In addition, Jiuzao is rich in bioactive components, including peptides, flavonoids, organic acids, and polyphenols, which have complex components [15]. These all indicate that prolamins might not be easy to extract from Jiuzao. It is reported that extraction efficiencies of kafirin from sorghum distillers dried grains were achieved at 44.2, 24.2, and 56.8% by using acetic acid, HCl-ethanol, and NaOH-ethanol, respectively [16]. It is also reported that the extraction efficiency of PBJ was attained at the highest (43.63%) when 70% ethanol (*w*/*w*) was used as the extraction solvent, followed closely by glacial acetic acid (40.11%), and 55% isopropanol had the lowest efficiency (32.11%) [1]. Despite the highest extraction efficiency, the PBJ extracted by 70% ethanol (*w*/*w*) had a darker color and lower purity than that via glacial acetic acid. Some impurities, such as polysaccharides, were easier to be co-extracted with 70% ethanol (*w*/*w*) [1]. In order to meet the requirements of forming nanocarriers to deliver bioactive compounds such as pharmaceutical molecules, a high purity of prolamins is needed. Therefore, combined approaches to improve extraction efficiency and purity of PBJ is necessary.

Reverse micelles (RMs) are self-assembly aggregates formed by surfactants in an organic solvent [17]. The nanometer-sized water pools formed in the polar cores of RMs can host various hydrophilic solutes, and provide a safe medium for bioseparation [18]. A typical reverse micelles (RMs) extraction includes two steps: forward and back extraction [19]. Highly efficient purification of the target molecules using RMs extraction can be achieved by varying parameters in both the organic phase and the aqueous phase, including pH, concentration of salts, ethanol volume fraction, and concentration of surfactant, etc. [20,21]. RMs extraction has been used to purify plant proteins such as soy protein [22], peanut protein [23], walnut protein [24], bromelain [25], and papain [26]. Therefore, RMs extraction has a high potential for downstream processing, especially in food science.

Some studies have found that there are conformational transformations of proteins purified by RMs extraction [23,27,28]. The proportion of β-sheet structure of 7S globulins from soybeans, prolamin, and glutelin fractions from walnut protein increased after bis (2-ethylhexyl) sulfosuccinate sodium salt (AOT) RMs extraction [23,28]. In our previous study, some of the disordered structures (random coil) of the microbial transglutaminase (MTGase) transformed into the ordered structures (α-helix) as the ionic strength increasing in CTAB backward extraction. It was also found that solvent polarity induces the original α-helices of prolamin (zein) to transform into β-sheet [29]. The change in the secondary structure of prolamin causes a difference in its self-assembly. Under this condition, the prolamin prefers to form a weak hydrophobic shell, a strong hydrophobic intermediate region, and a relatively stronger inner hydrophobic core, in which various bioactives are encapsulated [30].

The current research aims to use an NaOH-ethanol method and RMs extraction to obtain purified PBJ from Jiuzao. Secondary structures, morphology, and particle size distribution of PBJs were observed. This study aims to establish a technique combining NaOH-ethanol method and RMs extraction for prolamins recycled from Jiuzao.

## 2. Materials and Methods

### 2.1. Materials and Chemicals

Baijiu Jiuzao was obtained from Sichuan Wuliangye Co., Ltd. (Yibin, China). Cetyltrimethylammonium bromide (CTAB) was purchased from Aladdin Industrial, Inc. (Shanghai, China). BCA protein assay kit was purchased from Sangon Biotech Co., Ltd. (Shanghai, China). N-octane, ethanol, n-hexanol, and other chemicals were purchased from Titan Scientific Co. (Shanghai, China). All chemicals were of analytical grade.

### 2.2. Method

#### 2.2.1. Preparation of Crude PBJ Powder from Baijiu Jiuzao

The frozen Baijiu Jiuzao was dried at 80 °C until its mass was constant, then smashed and fitted with a 425 μm opening screen. The smashed powder of Jiuzao was extracted using NaOH-ethanol method according to the reference [1], and then defatted using n-hexane at 50 °C with a solvent-to-sample ratio of 10:1 (mL/g) three times to obtain crude PBJ powder.

#### 2.2.2. Forward Extraction

The extraction method was conducted according to the method described by previous references with some modifications [31]. A total of 1 mL 10–80% (*v*/*v*) ethanol solution (10 mM phosphate buffer (pH 4–9) containing 0.1–0.6 M GuHCl or KBr) and 1.0 mg crude PBJ powder was mixed with 1 mL of the CTAB/octane/hexanol solution (volume fraction of hexanol and octane was 1:5, [CTAB] = 20 mM. Vaq:Vrm = 1:1), followed by mixing for 5 min at 25 °C (XW-80A, Chitang Electronics Co., Ltd., Shanghai, China). The mixture was then centrifuged (TD5A, Xinchunlan Scientific Instrument Co., Ltd., Xiamen, China) at 5310× *g* for 20 min at 4 °C to achieve phase separation. The precipitate was discarded, and the organic phase (upper layer) collected from the forward extraction was subjected to the backward extraction.

#### 2.2.3. Backward Extraction

The stripping phase was prepared with ethanol (the volume fraction of ethanol was 30–80% (*v*/*v*)), 10 mM phosphate buffer (pH 5–11) containing 0.1–0.7 M KBr or chaotropes (urea and GuHCl). The organic phase (upper layer) collected from the forward extraction was mixed with equal volume of the stripping phase for 5 min, and then centrifuged at 5310× *g* for 10 min at 4 °C to achieve phase separation. The upper layer was discarded, the lower aqueous phase was collected and dialyzed at 4 °C for 24 h to remove impurities, salts, and excess surfactant. The water was refreshed three times daily. After dialysis, the protein solution was frozen at −20 °C overnight and then freeze-dried (FD-1C-50, Chudin Analytical Instrument Co., Ltd., Shanghai, China) for 48 h. The dried protein sample (purified PBJ powder) was stored at 4 °C for further analyses.

#### 2.2.4. Extraction Performance

The protein content in the aqueous and organic phases was measured using the BCA method according to references [23,31]. The protein extraction efficiency (%) was calculated using Equations (1) and (2).

Forward protein extraction efficiency (E_f_):

(1) $$E_{f}=\frac{C_{rm,f}\times V_{rm,f}}{M_{aq,0}}\times 100\%$$

Backward protein extraction efficiency (E_b_):

(2) $$E_{b}=\frac{C_{aq,b}\times V_{aq,b}}{C_{rm,f}\times V_{rm,f}}\times 100\%$$

M is the mass of crude PBJ powder added in forward extraction (mg), V is the volume of the solution (mL), C is the concentration of prolamins (mg·mL^−1^), aq is the aqueous phase, rm is the organic phase, 0 is the initial phase, f is forward extraction, and b is backward extraction.

#### 2.2.5. Fourier Transform Infrared (FTIR) Analysis

To characterize the structures and impurities of the PBJs, the purified PBJ (via further RMs extraction) and crude PBJ (via NaOH-ethanol method) powders were analyzed using an FTIR spectrometer (Frontier, PerkinElmer, Waltham, MA, USA) in potassium bromide tablets (spectrum pure). The absorbance spectra were obtained over the 4000–400 cm^−1^ region and with a nominal resolution of 4 cm^−1^ using 32 scans. The amide I band (1600–1700 cm^−1^) was processed with PeakFit (v4.12) software to obtain the secondary structures of PBJ in the solid-state.

#### 2.2.6. Scanning Electron Microscopy (SEM) Analysis

The morphology of the PBJ powders was observed using SEM (VEGA3, TESCAN, Brno, Czech Republic). Before SEM observation, a small amount of PBJs powder was spread onto an adhesive tape fixed onto a stainless-steel stub and coated with a layer of gold. The defined electron acceleration voltage is 15 kV. Magnification was chosen as 300×, 2000×, 5000×, and 10,000×. The software VegaTC was applied to analyze the SEM images.

#### 2.2.7. Dynamic Light Scattering (DLS) Analysis

The particle size distribution of the PBJs was measured using DLS method. The PBJ was dissolved into 10 mM phosphate buffer (pH 7.0) to prepare a solution with a concentration of 0.18 M. Then the particle size distribution of the PBJ was determined using a DLS microscope (Nano ZS90, Malvern, UK) with a JDS Uniphase He-Ne laser (vertically polarized beam, wavelength 632.8 nm).

#### 2.2.8. Statistical Analysis

All tests were carried out three times, and the mean values and the standard deviations were calculated. Data visualization was performed by using Origin software (v2021, academic applied). Significant differences among samples were determined using IBM SPSS Statistics 25 (SPSS, Chicago, IL, USA) (*p* < 0.05).

## 3. Results and Discussion

### 3.1. Reverse Micellar Extraction of PBJ from Baijiu Jiuzao

#### 3.1.1. Optimized Forward Extraction

**Figure 1:**
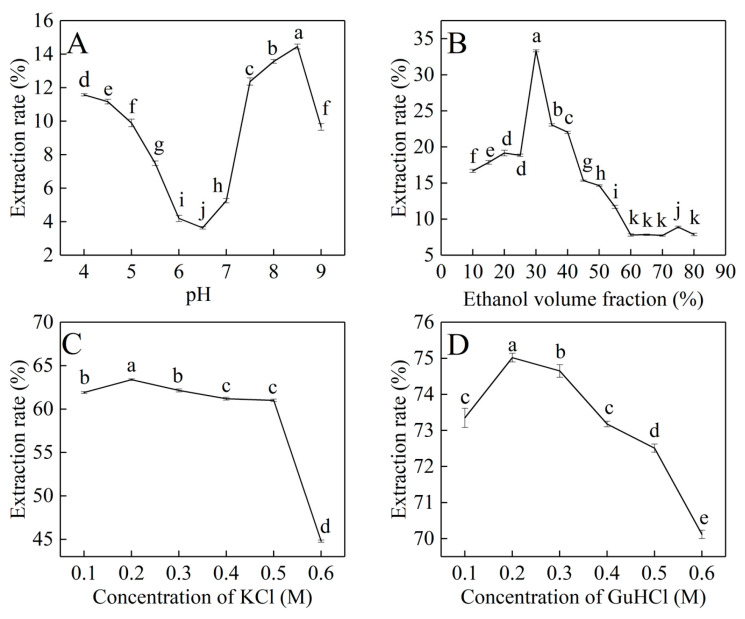
The effects of different factors on forward extraction efficiency of PBJ. (**A**) pH of the aqueous phase. (**B**) Ethanol volume fraction of the aqueous phase. (**C**) Concentration of KBr in the aqueous phase. (**D**) Concentration of GuHCl in the aqueous phase. Different letters above the lines and bars represent significant variations (*p* < 0.05).

The pH of the aqueous phase is one of the significant factors influencing the extraction efficiency [32]. As shown in Figure 1A, when the pH of the aqueous phase was adjusted from 4 to 9 (10 mM sodium phosphate buffer, with no salts and ethanol), the protein efficiency of forward extraction is nearly 15% at pH 8.5, which was the highest. So, the optimum pH for the PBJ forward extraction was pH 8.5.

Prolamins are plant-derived proteins that can be soluble in alcohol [33]. Since prolamin solubility in water is relatively low, in order to reduce the mass transfer resistance during forward extraction, ethanol was added into the aqueous phase. As shown in Figure 1B, when the volume fraction of ethanol in the aqueous phase was adjusted from 30% to 80% (10 mM sodium phosphate buffer, pH 8.5, with no salts), the protein efficiency of forward extraction was the highest, nearly 35%, when the volume fraction of ethanol is 30%. So, the optimum volume fraction of ethanol in the aqueous phase for the forward extraction of PBJs was 30%.

KCl is commonly used in reverse micelles extraction [34]. Figure 1C shows the effect of the concentration of KCl on the extraction of PBJ in forward extraction. The pH of initial aqueous feed was all adjusted to 8.5, and the volume fraction of ethanol was 30%. Figure 1C shows that the maximum efficiency (nearly 63%) was achieved when the concentration of KCl is 0.2 M. The extraction of PBJ increased slightly then decreased dramatically with the increasing salt concentration. Such behavior can be explained by a change in the size of the reverse micelles. Experimental data showed that the water content in organic phase decreases with an increase in salt concentration [35], which may cause a decrease in the reverse micellar size. This causes a size exclusion effect, thus it is difficult for the PBJ molecule to stay in reverse micelles. As the PBJ molecules release from reverse micelles, the protein efficiency of forward extraction decreases.

GuHCl was also added into the initial aqueous phase to achieve higher extraction efficiency. As shown in Figure 1D, the extraction efficiency of PBJ achieved 75.02% when the concentration of GuHCl was 0.2 M. This may be because it is advantageous to prevent the protein from denaturing under an alkaline pH condition when adding guanidium salt at a low concentration [36].

According to the experiment, the optimum extraction conditions of PBJ in CTAB forward reverse micelles extraction were as follows: pH was 8.5 (10 mM sodium phosphate buffer), ethanol volume fraction was 30%, GuHCl concentration was 0.20 M, and the extraction efficiency of PBJ under the optimum conditions was 75.02%.

#### 3.1.2. Optimized Backward Extraction

**Figure 2:**
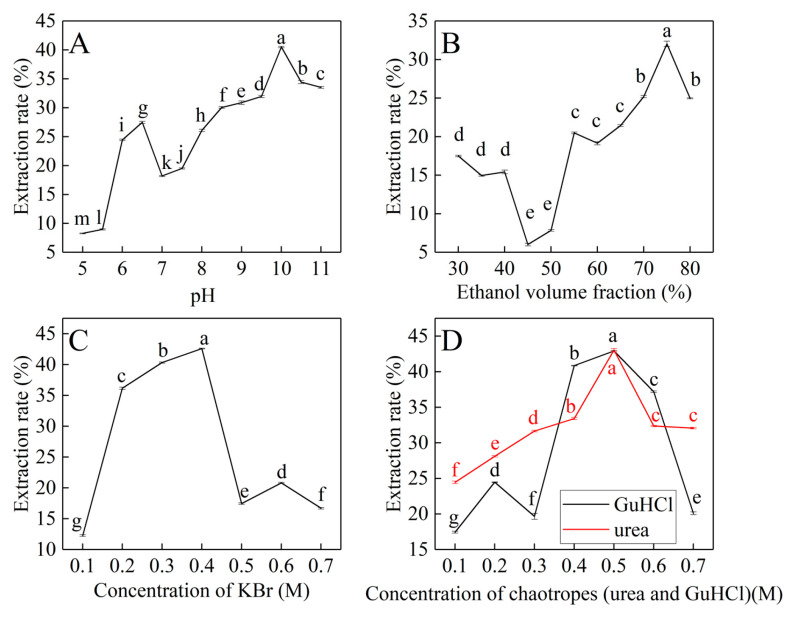
The effects of different factors on backward extraction efficiency of PBJ. (**A**) Ethanol volume fraction of the stripping solution. (**B**) pH of the stripping solution. (**C**) Concentration of KBr in the stripping solution. (**D**) Concentration of chaotropes (GuHCl and urea) in the stripping solution. Different letters above the lines and bars represent significant variations (*p* < 0.05).

The backward extraction parameters, including the pH, ethanol volume fraction, ionic strength, and chaotropes concentration in the stripping solution were studied. Figure 2A shows the effect of the volume fraction of ethanol in the stripping solution on the extraction of PBJ in backward extraction. When the volume fraction of ethanol was 75%, the extraction efficiency reached nearly 32%. Since prolamin is hydrophobic, which prefers to dissolve in organic solvents, increasing volume fraction of ethanol in the stripping solution makes an effort to achieve relatively high extraction efficiency of prolamins in backward extraction.

Figure 2B shows the effect of pH on the PBJ backward extraction. It can be seen that the extraction efficiency of PBJ increased with increasing pH value in the stripping solution. Almost 40% of PBJ transferred into the aqueous phase after backward extraction at pH 10.

As shown in Figure 2C, the extraction efficiency of PBJ first increased and then decreased with increasing concentration of KBr (0.1–0.7 M). Such behavior can be explained in terms of two effects: salting-in and size exclusion. With the increase in ion strength at a low concentration range (0.1–0.2 M), salting-in effect occurs, so that the solubility of PBJ in the stripping solution increases. Also, the increase in the ionic strength leads to a decrease in the size of reverse micelles [37], which causes the size exclusion effect where the protein is “squeezed” out of the reverse micelles. It is precisely because of these two reasons that the extraction efficiency of PBJ backward extraction increased.

Figure 2D shows the extraction efficiency of PBJ with increasing chaotropes (urea and GuHCl) concentration. When adding chaotropes at a low concentration in the stripping solution, water content in reverse micelles decreased [38], and the size of reverse micelles reduced, causing a reduction in extraction efficiency of backward extraction. Hence, according to the obtained results (Figure 2D), the maximum backward extraction efficiency (E_b_) of PBJ is 43.04% at 0.5 M urea (pH 10, 10 mM sodium phosphate buffer, volume fraction of ethanol is 75%), which was slightly higher than 42.96% at 0.5 M GuHCl.

Therefore, the optimum extraction conditions of PBJ in CTAB backward reverse micelles extraction were as follows: pH was 10 (10 mM sodium phosphate buffer), ethanol volume fraction was 75%, urea concentration was 0.50 M, and the extraction efficiency of PBJ under the optimum conditions was 43.04%.

The total extraction efficiency is the product of the forward extraction efficiency (75.02%) and the backward extraction efficiency (43.04%). Thus, the total extraction efficiency of PBJ is 32.29%. It is reported that the total extraction efficiency of bromelain from pineapple peel wastes using gemini surfactant-based RMs extraction was 59% [19]. The AOT RMs extraction efficiency of hemp protein from defatted hemp flours was 52.44% [31]. The main reason why the total extraction efficiency of PBJ is lower than that of bromelain and hemp protein may be that the surfactant CTAB and its concentration (0.02 M) are not the most suitable conditions for RMs extraction of PBJ.

### 3.2. Appearance of PBJ Powders

**Figure 3:**
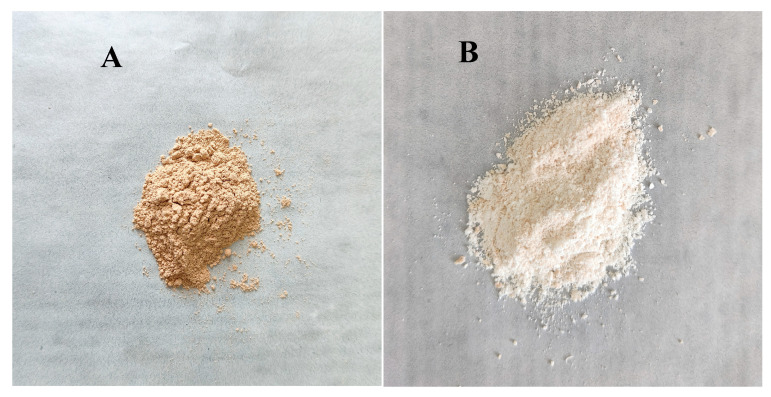
Appearance of PBJ powders. (**A**) Crude PBJ obtained by NaOH-ethanol method; (**B**) purified PBJ obtained by further RMs extraction.

There were large differences between the colors of crude and purified PBJ powder (Figure 3). The crude PBJ obtained by NaOH-ethanol method had a darker color compared with the purified ones obtained by further RMs extraction. It is reported that the PBJ extracted by glacial acetic acid (Lab = 79.99/1.01/13.20) with the lightest color had the highest purity, 98.36%, followed by the PBJ extracted by 70% ethanol (*w*/*w*, Lab = 55.79/7/28.12) with a light color and 55% isopropanol (*w*/*w*, 63.96/6.24/29.01) with a dark color, which had 93.65% and 62.42% purity, respectively [1]. The color differences demonstrated the different purities of the products [1]. The lighter the color, the higher the purity. Thus, the purified PBJ with a lighter color may have higher purity than the crude ones.

### 3.3. FTIR Curve-Fitting Analysis of PBJ

**Figure 4:**
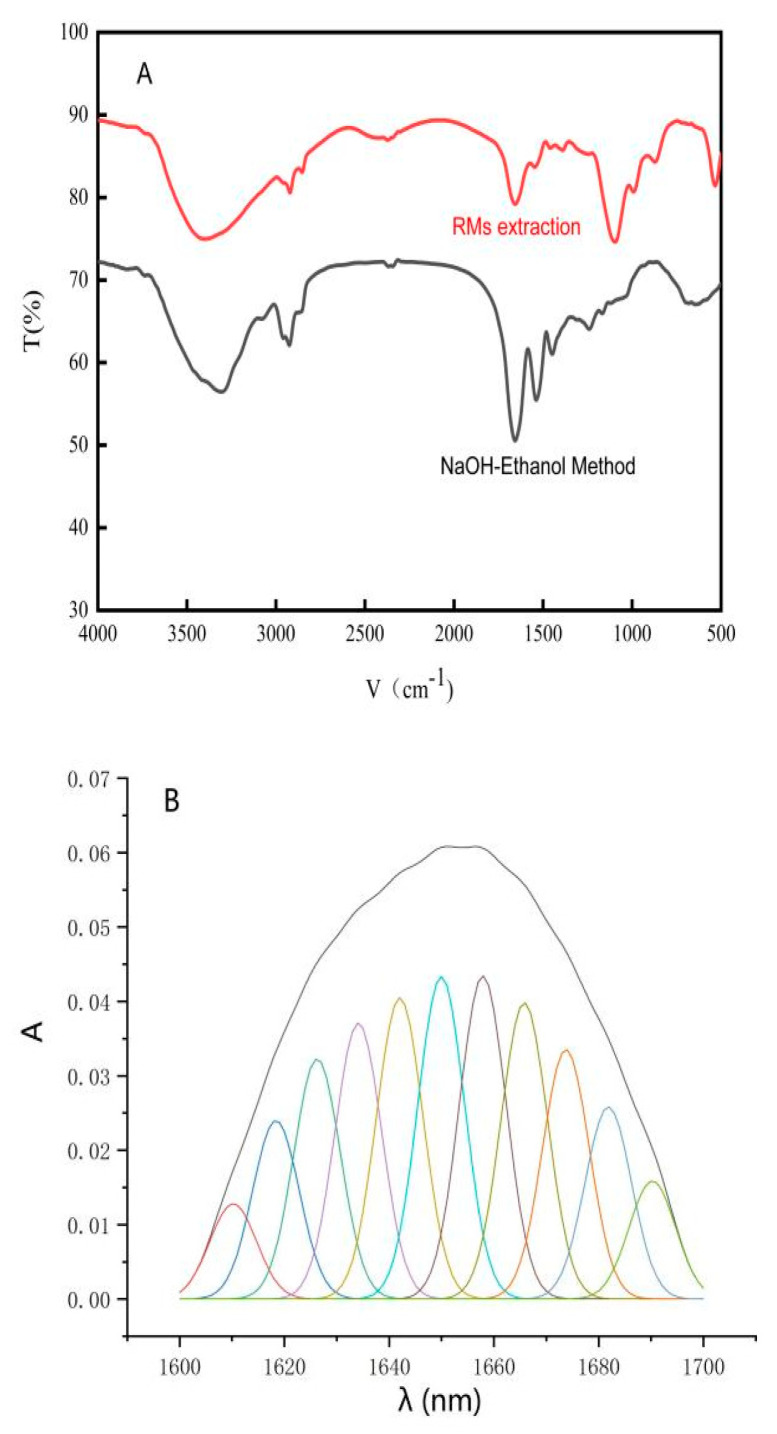

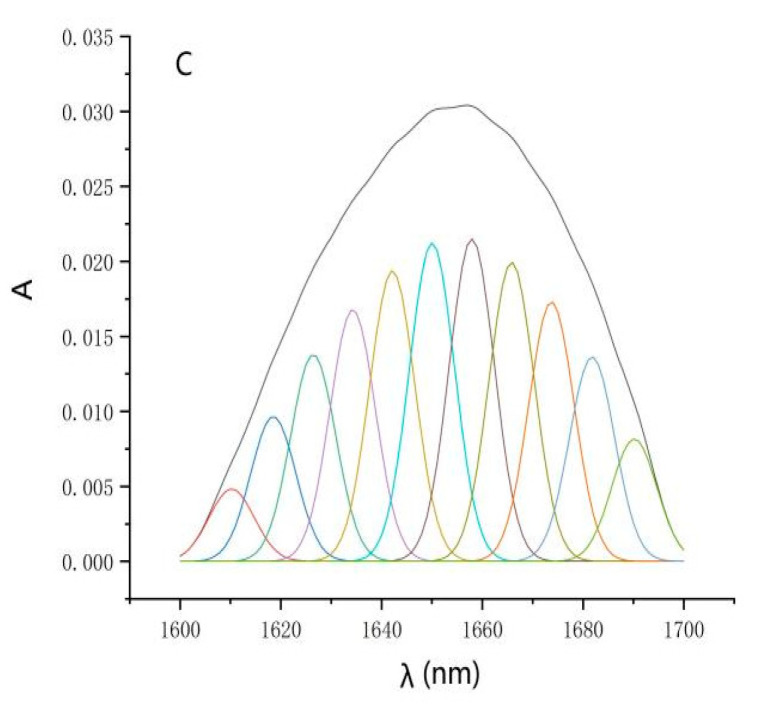
(**A**) FTIR spectra of PBJ. (**B**) The best fit for the amide I bands of the FTIR spectrum of crude PBJ obtained by NaOH-ethanol method. (**C**) The best fit for the amide I bands of the FTIR spectrum of purified PBJ obtained by further RMs extraction. A represents absorbance. Different colors represent that the peaks has different locations.

FTIR is an efficient technique to analyze the second structure of proteins with single sample preparation. Furthermore, FTIR is also a relevant technique that reveals the chemistry of prolamins leading to the necessary single biomolecular interactions [39] to develop smart encapsulation systems [40]. For quantitative determination of secondary structural changes between purified PBJ obtained by further CTAB RMs extraction and crude PBJ obtained by NaOH-ethanol method, curve-fitting analysis was used to the amide I region (1700–1600 cm^−1^). The original amide I region spectra and the deconvoluted spectra of PBJ are shown Figure 4A. The corresponding peaks of the fitted bands were assigned according to references [40], as shown Table 1. The band numbers separated from the deconvoluted spectra of purified PBJ obtained by further RMs extraction and crude PBJ obtained by NaOH-ethanol method were both 11 (Figure 4B,C). For β-sheet and turn structures, the band numbers in PBJ did not change (Table 1). For the unordered structure, the band number in purified PBJ decreased by 1, compared with that in crude PBJ. Meanwhile, for α-helix structure the band number in purified PBJ increased by 1. The frequencies at 1649 cm^−1^ shifted to a little bit higher position at 1650 cm^−1^, which indicated that the unordered structure conversed into α-helix structure. This may be the result of conformation changes of proteins in a different microenvironment [23].

### 3.4. Quantification Assay of Secondary Structures from PBJ

Significant differences (*p* < 0.05) between sample points for different lowercase letters in the upper-right corner of the data are shown in the same column as Table 1.

The quantity of peak area and contents of the corresponding protein secondary structure are given in Table 2. The percentage of α-helix structure in purified PBJ from further RMs extraction, compared with that in crude PBJs from NaOH-ethanol method, was significantly increased by 13.25 percentage points. The conformation changes of proteins may be caused by the electrostatic field between the enzyme molecule and the surfactant head-groups [41]. There are some other cases reported that the percentage of α-helix structure in protein increased after RMs extraction [42]. This indicated that RMs extraction purification may increase the α-helix structure percentage in protein.

The percentage of β-sheet structures in crude PBJ obtained by NaOH-ethanol method from 30.48% to down to 27.12% in purified PBJ obtained by further RMs extraction. This is while the percentage of β-turn structure in crude PBJ increased from 33.01% to up to 35.52% in purified PBJ. The reasons probably were attributed to the fact that the water pool in reverse micelle system caused a greater hydration of proteins dissolved in, which would further increase the proportion of turn structure at the cost of a reduction in β-sheet content [43].

The purified PBJ obtained by further RMs extraction showed a lower percentage of unordered structures compared with the crude PBJ. This result suggested that the reverse micelle system might increase the α-helix structure percentage in protein, and had not completely destroyed the secondary structures of PBJ [44]. Since the surface hydrophobicity of proteins and helical content has a negative correlation [45], thereby a higher α-helix content in the purified PBJ indicated less hydrophobic side chains were exposed on the protein surface. Compared with the crude PBJ, these purified PBJs with a higher proportion of α-helix and less hydrophobicity may have a higher solubility in water.

### 3.5. The Morphology of PBJ

**Figure 5:**
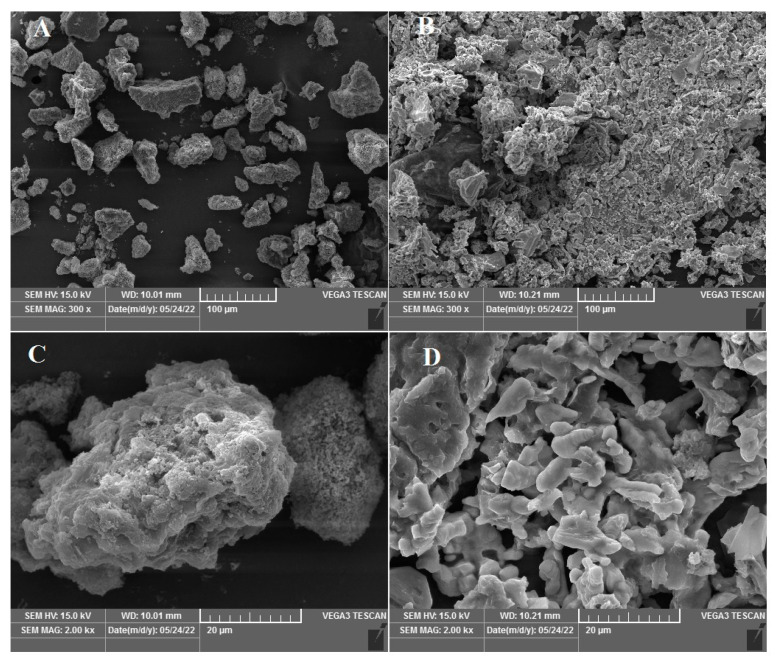

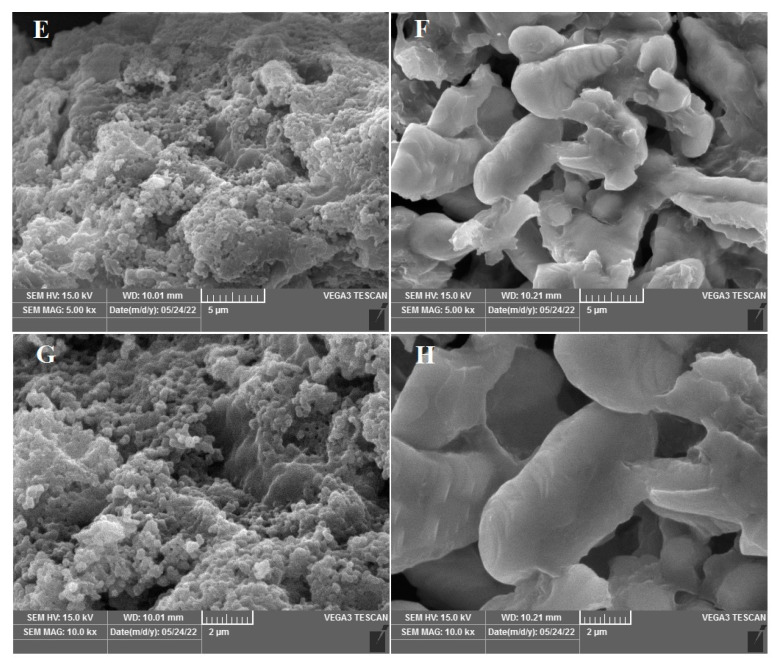
SEM image of PBJ powder. SEM images of crude PBJ powder obtained by NaOH-ethanol method, (**A**) (300×), (**C**) (2000×), (**E**) (5000×), (**G**) (10,000×). SEM image of purified PBJ powder obtained by further RMs extraction, (**B**) (300×), (**D**) (2000×), (**F**) (5000×), (**H**) (10,000×).

The surface morphology of the PBJ powder was observed by SEM. Six selected vision fields of SEM images were collected, showing the surface morphology of the PBJ powder (Figure 5). Under 300×, 2000× magnification, the crude PBJ powder obtained by NaOH-ethanol method formed nano-particles of about 10–100 nm in diameter. Under 5000×, 10,000× magnification, the crude PBJ powder had a rough surface consisted of even globose or dumbbell particles, which was similar to PBJ powder extracted by acetic acid [1].

Under 300×, 2000× magnification, compared with the crude PBJ powder via NaOH-ethanol method, the purified PBJ powder via further RMs extraction formed a complex protein network, illustrated by a big chunk of flaky texture with small pores. Under 5000×, 10,000× magnification, the purified PBJ powder consisted of flake or dumbbell particles, which had smooth surfaces. It indicated that after further purification the PBJ powder contained less impurities, thus during freeze-dry the PBJ protein molecules could congregate to form a complex large-scale network without disturbance of impurity molecules.

### 3.6. The Particle Size Distribution of PBJ

**Figure 6:**
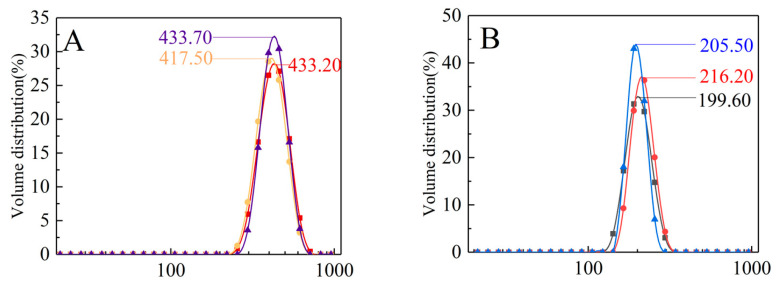
Size of PBJ in the solution state. (**A**) The crude PBJ obtained by NaOH-ethanol method. (**B**) The purified PBJ obtained by further RMs extraction.

DLS was applied to study the particle size distribution of PBJ in the solution state (Figure 6). As shown in Figure 6, purified PBJ obtained by further RMs extraction had narrower particle size distribution, compared with the crude PBJ obtained by NaOH-ethanol method, which indicated that the purified PBJ was more uniform. The average particle size of crude PBJ obtained by NaOH-ethanol method was 428.13 ± 9.21 nm (Figure 6A), while the average particle size of purified PBJ obtained by further RMs extraction was 207.10 ± 8.41 nm (Figure 6B). The average particle size of purified PBJ is remarkably smaller (*p* < 0.01) than those of crude PBJs. The reduction in the average particle size of PBJ might be attributed to the changes in the secondary structures of purified PBJ after RMs extraction. The α-helix structure percentage of purified PBJs by further RMs extraction was significantly increased by 13.25%, while unordered structure percentages decreased by 12.49%, leading to more compactness of prolamin molecules and a lesser molecule size.

## 4. Conclusions

In summary, this work recycled prolamin from Baijiu Jiuzao via NaOH-ethanol method and further RMs extraction, and characterized the morphology, secondary structure, and particle size distribution of PBJ. Impact factors such as the pH, ethanol ratio, concentration of salts, and chaotropes were investigated and compared to confirm the optimum conditions of RMs extraction. The forward extraction efficiency of PBJ achieved 75.02%, and the backward extraction efficiency of PBJ was 43.04%. Purified PBJ powder obtained by further RMs extraction had a lighter color than crude PBJ powder obtained by NaOH-ethanol method, which indicated a higher purity of the purified PBJ. Morphology analysis showed that the purified PBJ formed a larger-scale complex protein network than the crude PBJ. The percentages of α-helix and β-turn structures in purified PBJ was higher than that in the crude PBJ, while the percentages of β-sheet and unordered structures were lower. This result indicated an increase in compactness of PBJ molecules after further RMs extraction, which was proved by the DLS analysis. The average particle size of PBJs in the solution state decreased from 428.13 ± 9.21 (the crude PBJ) nm to 207.1 ± 8.41 (the purified PBJ) nm. Compared with the crude PBJ, the purified PBJ with a higher proportion of α-helix and smaller average particle size may have better water-solubility and application prospects in food and feed additives.

The findings from the current study reveal that the reverse micelles have potential to be a promising tool to recycle and modify the prolamin from Baijiu Jiuzao, and this will promote the application of PBJ in the food and pharmaceutical industry. Further investigation into the relationship of structure and functional properties of PBJ should be carried out to improve functionality and industrial targets to develop products.

## Figures and Tables

**Table 1:** Amide I frequencies for PBJ. Crude PBJ was obtained by NaOH-ethanol method, and purified PBJ was obtained by further RMs extraction.

Extraction Method	Deconvoluted Spectrum (cm^−1^)			
	β-Sheet (1600–1640 cm^−1^)	Unordered (1640–1650 cm^−1^)	α-Helix (1650–1660 cm^−1^)	Turn (1660–1700 cm^−1^)
NaOH-ethanol Method	1610, 1618, 1626, 1634	1642, 1649	1658	1666, 1674, 1682, 1690
Further RMs Extraction	1610, 1619, 1626, 1634	1642	1650, 1658	1667, 1674, 1682, 1690

**Table 2:** Content of the secondary structure in PBJs by FTIR analysis.

Extraction Method	Secondary Structure (%)			
	α–Helix	β–Sheet	Unordered	Turn
NaOH-Ethanol Method	12.46 ± 0.91 ^c^	30.48 ± 1.13 ^a^	24.15 ± 0.44 ^b^	33.01 ± 3.79 ^a^
Further RMs Extraction	25.71 ± 1.28 ^b^	27.12 ± 1.18 ^b^	11.66 ± 1.43 ^c^	35.52 ± 1.02 ^a^
Differences	13.25	−3.36	−12.49	2.51

Different letters in the upper-right corner of the data represent significant variations (*p* < 0.05).

## Data Availability

The original contributions presented in the study are included in the article, further inquiries can be directed to the corresponding author.
